# Comparative Transcriptomic Profiling in Patients Affected by Duchenne and Becker Muscular Dystrophies: A Focus on ECM Genes Dysregulation

**DOI:** 10.3390/ijms26146594

**Published:** 2025-07-09

**Authors:** Bartolo Rizzo, Francesca Dragoni, Maria Irene Dainesi, Rosalinda Di Gerlando, Evelyne Minucchi, Angela Lucia Berardinelli, Stella Gagliardi

**Affiliations:** 1 IRCCS Mondino Foundation, 27100 Pavia, Italy; b.rizzo@golgicenci.it (B.R.); mariairene.dainesi01@universitadipavia.it (M.I.D.); rosalinda.digerlando@mondino.it (R.D.G.); evelyne.minucchi@mondino.it (E.M.); angela.berardinelli@mondino.it (A.L.B.); stella.gagliardi@mondino.it (S.G.); 2 Golgi Cenci Foundation, 20081 Abbiategrasso, Italy; 3 Department of Brain and Behavioural Sciences, University of Pavia, 27100 Pavia, Italy; 4 Department of Biology and Biotechnology “Lazzaro Spallanzani”, University of Pavia, 27100 Pavia, Italy

**Keywords:** Duchenne muscular dystrophy, Becker muscular dystrophy, ECM, RNA-seq, collagen, fibrosis, inflammation, skeletal muscle

## Abstract

The complexity of RNA metabolism has become crucial in neuromuscular diseases, especially for Duchenne muscular dystrophy (DMD) and Becker muscular dystrophy (BMD). Our goal was to search for possible pathways that differ between the two diseases, in which DMD develops a severe phenotype compared to BMD. In this work, we aimed to evaluate the transcriptomic profile in skeletal muscle biopsies derived from patients with either DMD or BMD. We collected RNA obtained from pediatric patients with DMD (n = 12) and with BMD (n = 6). Compared to patients with BMD, patients with DMD showed a particular activation of genes involved in collagen synthesis, extracellular matrix organization, and Oncostatin M-dependent pathways, important for fibrotic processes. This suggests that a more severe phenotype in patients with DMD compared to those with BMD may be due to greater deregulation of these pathways, reflecting the clinical picture of patients observed. Our results allowed us to highlight the molecular differences between the two phenotypic groups, shedding light on the pathways that make Duchenne dystrophy more severe than its counterpart does. This study provides preliminary insights into the difference in gene expression between the two groups and lays the basis for the identification of possible mechanisms that differentiate between the two diseases.

## 1. Introduction

Muscular dystrophies are a group of severe and progressive diseases characterized by muscle atrophy [1]. Duchenne muscular dystrophy (DMD) and its milder variant, Becker muscular dystrophy (BMD), are the most common forms in childhood [2,3]. DMD is caused by mutations in the *DMD* gene, which encodes dystrophin. These mutations prevent the production of the full-length muscle isoform of the protein (Dp427m) [1], which is critical for maintaining the strength and stability of muscle fibers [4]. Mutations in the same gene can also lead to BMD, a milder form with later onset and slower progression than DMD [1]. The two conditions can be genetically distinguished by the type of mutation: DMD is primarily caused by out-of-frame mutations, while BMD is usually due to in-frame mutations [5]. In both cases, the loss of dystrophin results in muscle atrophy of varying severity, along with respiratory and cardiac complications [6].

The absence of dystrophin disrupts the dystrophin–glycoprotein complex (DGC), leading to membrane instability and increased susceptibility to mechanical damage and fiber necrosis. Moreover, patients with DMD show impaired regenerative capacity of myofibers, leading to the replacement of muscle tissue with fibro-fatty tissue [7]. In contrast, patients with BMD typically harbor mutations that maintain the reading frame, allowing the production of truncated yet partially functional dystrophin proteins [8].

Clinically, dystrophinopathies are characterized by muscle weakness predominantly affecting the limb–girdle regions, muscle pseudo hypertrophy, progressive degeneration, and possible cardiac and respiratory failure. The central nervous system (CNS) is also progressively affected in DMD, and developmental delays, speech difficulties, and neuropsychiatric traits such as autism spectrum behaviors and obsessive–compulsive tendencies are often observed [9]. According to classical definitions, clinical onset in DMD occurs in early childhood, followed by progressive skeletal muscle weakness that typically results in loss of ambulation during the second decade of life. Cardiorespiratory complications emerge in the third decade, significantly affecting life expectancy and quality of life. BMD typically presents later—around 8 years of age—and progresses more slowly, with ambulation preserved for a longer period. Intermediate or milder phenotypes between DMD and BMD may also result from other mutations in the *DMD* gene [10].

In both DMD and BMD, dystrophin deficiency leads to the disruption of the dystrophin-associated glycoprotein complex (DAPC) and a loss of connectivity between F-actin and the extracellular matrix (ECM). DAPC disruption adversely affects muscle cell function by weakening the sarcolemma and contributing to functional ischemia, oxidative stress, cytosolic calcium overload, impaired muscle regeneration, and cardiomyopathy, as well as cognitive deficits [11,12]. Defects in the contractile apparatus perpetuate inflammatory responses, which in DMD become chronic and play a key role in disease progression [13]. Accumulation of pro-inflammatory mediators in skeletal muscle further impairs regeneration and promotes fibrosis and excessive ECM deposition, ultimately leading to functional muscle loss [14,15,16,17].

From a molecular and biological standpoint, numerous transcriptomic studies have been conducted on both animal [18,19,20] and human models [21,22], providing insights into the roles of deregulated RNAs in muscle stem cell quiescence, proliferation, and differentiation during myogenesis [22]. Spatial and single-cell transcriptomic analyses have revealed the strong involvement of genes associated with muscle regeneration [19], fibrosis (e.g., *VIM*, *FN1*, *THBS4*), and calcification (e.g., *BGN*, *CTSK*, *SPP1*), as well as the role of macrophages in ECM remodeling and tissue repair. Heezen and colleagues identified specific cell clusters and gene expression signatures linking transcriptomic alterations with histological changes in DMD mouse model tissues [19]. RNA-seq analyses have also shown increased expression of collagen genes (e.g., *COL1A1*) and other ECM components, contributing to skeletal muscle fibrosis [21].

In terms of RNA metabolism, research has increasingly focused on the molecular mechanisms that regulate mRNA translation, which are critical for skeletal muscle adaptation to environmental and physiological stimuli such as exercise, diet, and disease [23]. While many skeletal muscle adaptations are driven by transcriptional changes, post-transcriptional regulatory mechanisms also play essential roles in modulating muscle physiology [24]. These RNA regulatory pathways, including those involved in regeneration, are disrupted in various muscular dystrophies. Nieves and colleagues, through RNA-sequencing analysis, demonstrated that calcium homeostasis, ECM remodeling, and apoptotic pathways are significantly affected in dystrophic muscles [25].

Given the relative lack of transcriptomic data specifically comparing BMD and DMD, this study aimed to analyze the gene expression profiles of skeletal muscle biopsies from six patients with BMD and twelve with DMD. Our goal was to identify molecular signatures that might explain the differences in disease severity and progression observed between these two forms of dystrophinopathy.

## 2. Results

We performed RNA-seq analysis to investigate the expression of coding and long non-coding RNAs in muscle biopsies from patients with DMD or BMD. Only transcripts with |log_2_FC (DMD sample/BMD sample)| ≥ 1 and a false discovery rate (FDR) ≤ 0.1 were retained for further analysis.

### 2.1. Overview of Differentially Expressed (DE) Genes

A total of 2215 differentially expressed (DE) genes were identified. Of these, 1809 were protein-coding genes, including 354 downregulated and 1455 upregulated genes. Additionally, 406 DE genes were non-coding, with 268 upregulated and 138 downregulated; however, these non-coding genes were not the focus of the present study. DE genes in each category are reported in Table 1, along with their regulation status (up or down). The “non-coding” column includes different biotypes of non-coding RNAs (e.g., processed pseudogenes, processed transcripts).

**Figure 1:**
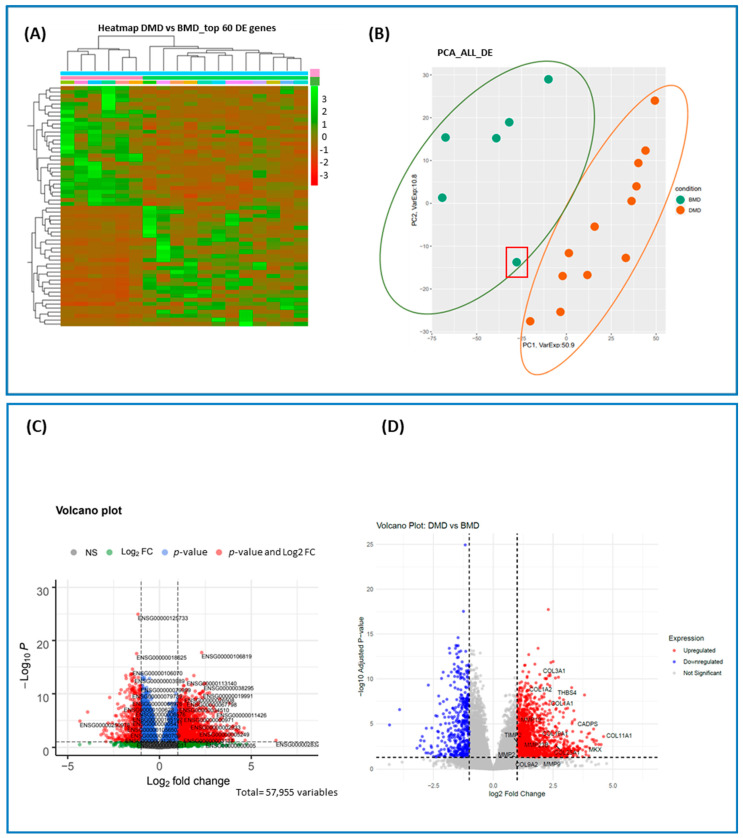
Differential expression analysis in biopsies of skeletal muscle of DMD patients and BMD patients. (**A**) Heatmap representing the expression profiles of DE genes in DMD patients (light green bar) and BMD patients (pink bar). (**B**) Principal component analysis (PCA) of all DE genes. PC1: 50.9 (*x*-axis) and PC2: 10.8 (*y*-axis). The DMD group is represented in orange and the BMD group in green. Only one sample seems to have an RNA profile in between DMD and BMD (red box). The graph shows the top 60 genes that are expressed with opposite trends between the two groups (50% of genes that are upregulated in BMD subjects (upper left side in green), are downregulated in DMD subjects (upper right side in red) and 50% of genes that are downregulated in BMD patients (lower left side in red), are upregulated in DMD subjects (lower right side in green). (**C**,**D**) Volcano plots. DE genes in DMD patients compared to BMD patients. The expression difference is considered significant for a log2 fold change of ≥1 or ≤−1 (*x*-axis) and for false discovery rate ≤ 0.1 (*y*-axis). Red dots represent significantly up- and downregulated genes that have |log_2_(fold change)| ≥ 1 and a *p*-value ≤ 0.05. Green dots represent significantly up- and downregulated genes that have |log_2_(fold change)| ≥ 1. Blue dots represent significantly up- and downregulated genes that have a *p*-value ≤ 0.05. Grey dots represent detected DE genes that are not significant, because they do not satisfy both requirements. (NS = nonsignificant; log_2_FC = satisfying fold change criteria; P: satisfying *p*-value criteria; P and log2FC: satisfying both fold change and *p*-value cut-off). (**C**) The top DE genes are labeled (Ensembl ID). (**D**) The main 10 ECM-related DE genes are labeled with their GeneCard name.

We compared the top 60 DE genes between the DMD and BMD patient groups by generating a heatmap to visualize differences in gene expression profiles (Figure 1A). As shown, the gene expression patterns clearly distinguish the DMD group from the BMD group, with a marked separation and opposite expression trends.

To enhance interpretability and minimize information loss, we performed principal component analysis (PCA) on all DE genes (Figure 1B). This analysis confirmed a clear separation between the DMD and BMD groups. Notably, one sample exhibited an RNA profile intermediate between the two groups (highlighted in the red box). Interestingly, this BMD patient appears to have a more compromised transcriptomic profile, which could be described as “intermediate” between typical BMD and DMD features.

Furthermore, volcano plots were constructed separately for each group to highlight all statistically significant DE genes (Figure 1C,D). Figure 1D specifically highlights key extracellular matrix (ECM)-related DE genes identified in the RNA-seq analysis.

### 2.2. Gene Set Enrichment Analysis

**Figure 2:**
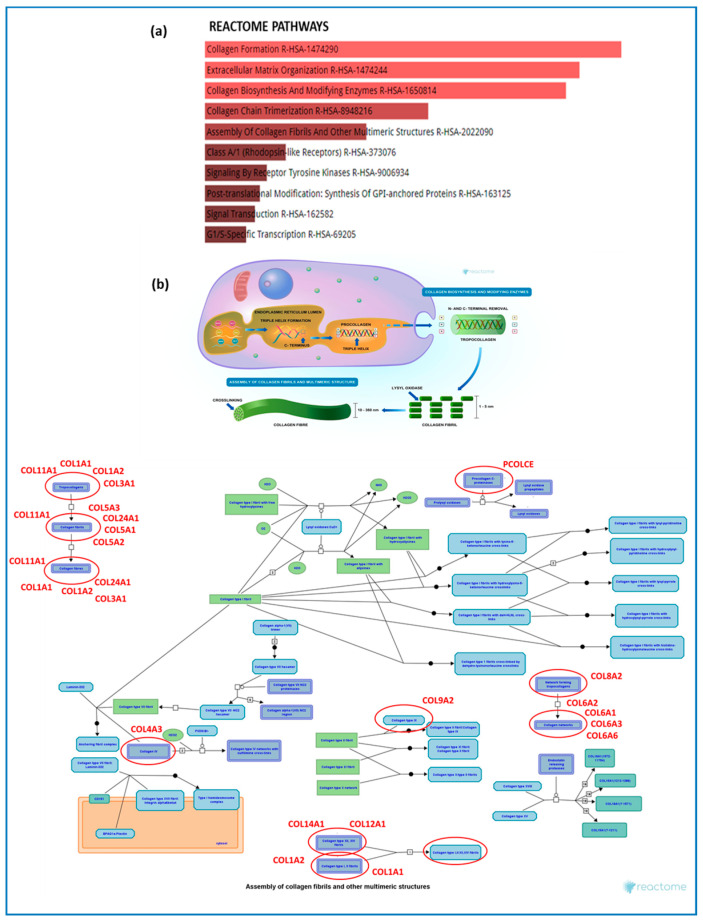
Enrichment pathway analysis. (**a**) Reactome most enriched pathways between patients with DMD and with BMD. (**b**) Reactome image of the first enriched pathways emerging from the analysis with deregulated collagen genes marked in their environment (red circle and name). For clearer image please visit: https://reactome.org/PathwayBrowser/#/R-HSA-1650814&PATH=R-HSA-1474244,R-HSA-1474290&DTAB=MT (accessed on 15 February 2025).

Reactome pathway analysis revealed increased deregulation of processes involved in collagen formation, extracellular matrix organization, biosynthesis, and collagen chain trimerization in DMD patients compared to BMD patients (Figure 2a,b).

**Figure 3:**
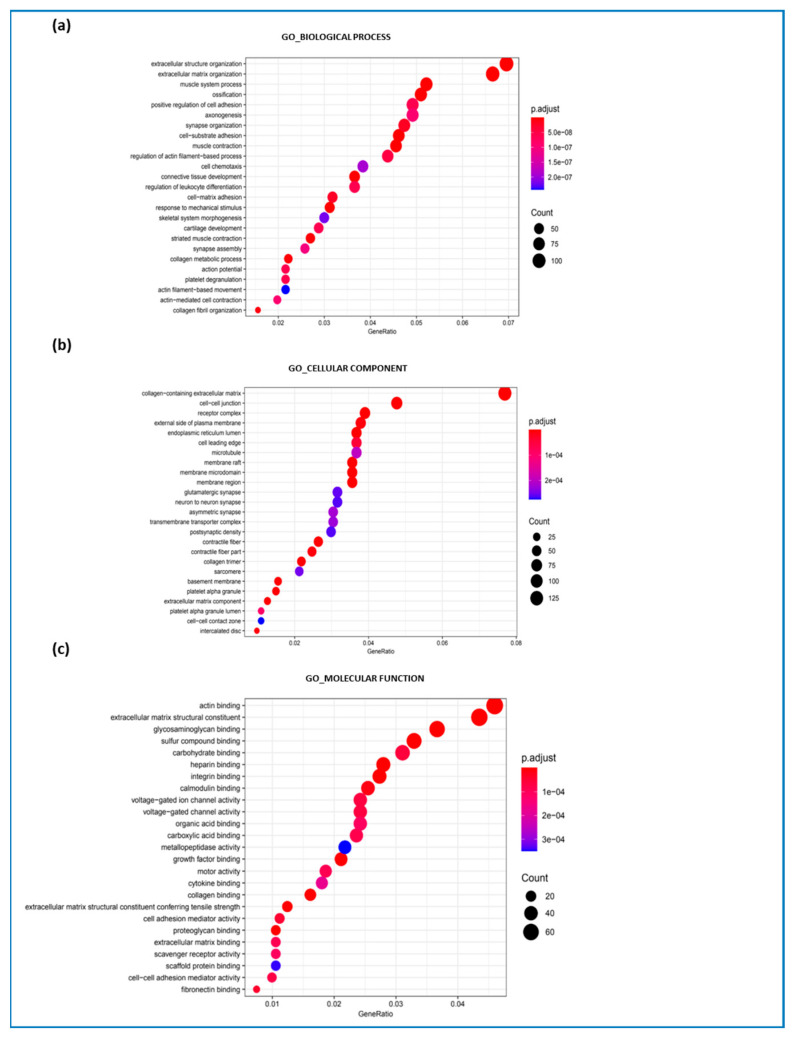
Gene ontologies. (**a**) DMD vs. BMD GO-enriched terms for biological processes, (**b**) cellular components, and (**c**) molecular functions.

In addition, GO biological process (Figure 3a), GO cellular component (Figure 3b), and GO molecular function (Figure 3c) analyses were performed, comparing patients with DMD and with BMD. GO biological process analysis showed that processes involved in ECM organization, extracellular structure organization, and muscle system processes were deregulated in DMD compared to BMD (Figure 3a). Consequently, significant alterations in collagen activity and extracellular matrix components led to the involvement of cellular components (Figure 3b), primarily affecting collagen within the extracellular matrix and cell–cell junctions. Regarding deregulated GO molecular functions, actin binding, extracellular matrix structural constituents, and glycosaminoglycan binding were largely implicated (Figure 3c). Furthermore, deregulated pathways related to synapse organization, axonogenesis, action potential, and synapse assembly were observed among the altered biological processes. Pathways associated with neuron-to-neuron communication, asymmetric synapses, and glutamatergic synapses were also deregulated in the context of cellular components. Another interesting set of deregulated pathways was linked to the inflammatory response, including regulation of leukocyte differentiation and platelet activation, which relate to vascular abnormalities (Figure 3a,b).

### 2.3. mRNA Expression Levels of the Most Differentially Expressed Genes Between DMD and BMD Patients

**Figure 4:**
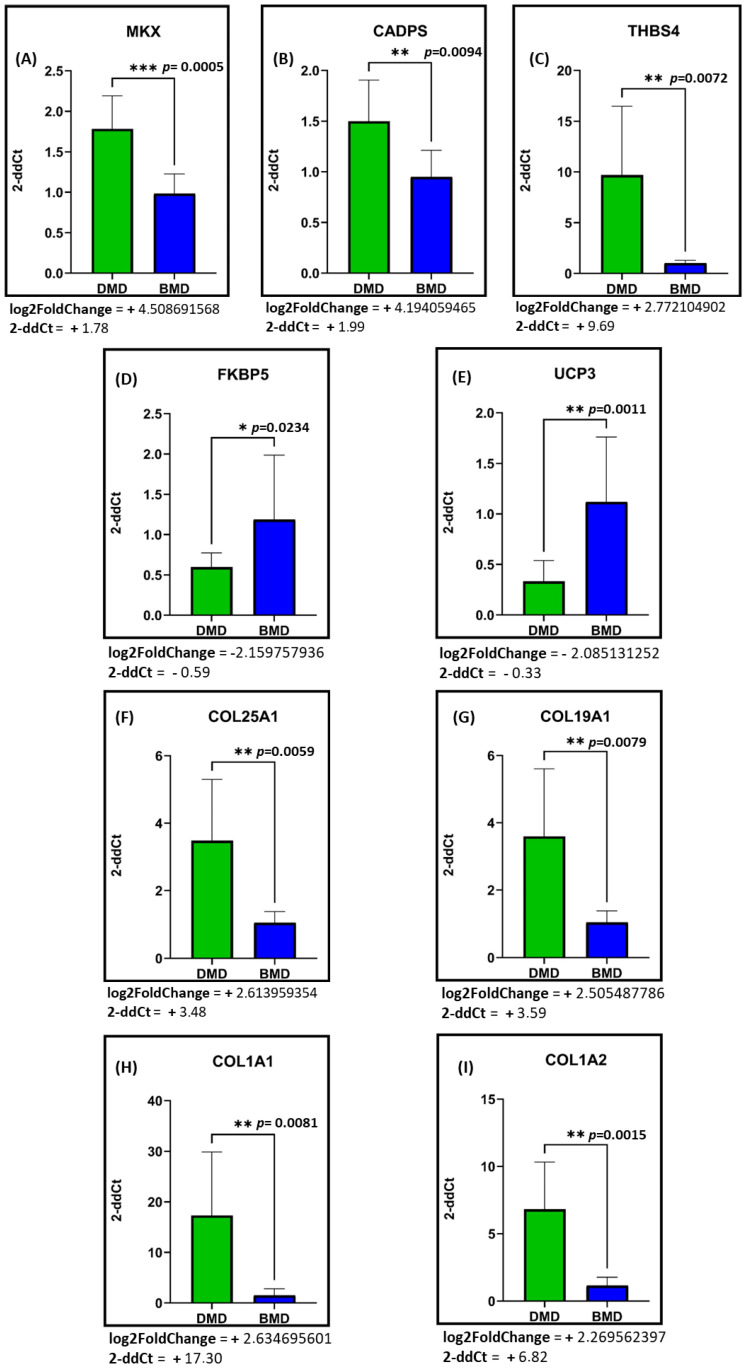
mRNA expression levels of DE genes among Duchenne versus Becker patients. RT-qPCR of (**A**) *MKX*; (**B**) *CADPS*; (**C**) *THBS4*; (**D**) *FKBP5*; (**E**) *UCP3*; (**F**) *COL25A1*; (**G**) *COL19A1*; (**H**) *COL1A1*; and (**I**) *COL1A2*. For each gene, we performed the analysis comparing the two pathologies. DMD patients n = 12; BMD patients n = 6. Cycle threshold (Ct) values were automatically recorded for each replicate qPCR reaction, and mean Ct values were normalized against those determined for *GAPDH* (Housekeeping Gene). Fold-expression differences were determined using the 2-ddCt method. Each gene has been evaluated in technical duplicate and each patient represents a biological replicate for the specific pathological condition. Under each graph, we also report values for the Log_2_FoldChange of validated genes derived from RNA-seq analyses, and 2-ddCt values relative to DMD patients compared to BMD patients. All the expected expression trends are confirmed. Statistical analysis was performed using the Unpaired Student’s *t*-test and data are expressed by means ± SEM. Two-tailed *p*-value. * *p* < 0.05, ** *p* < 0.01, *** *p* < 0.001.

We validated by RT-qPCR the expression levels of nine DE genes (*MKX*, *CADPS*, *THBS4*, *UCP3*, *FKBP5*, *COL1A1*, *COL1A2*, *COL25A1*, and *COL19A1*). For RT-qPCR validation, we used the RNA extracted from the same muscle biopsies derived from the BMD and DMD cohorts of patients used for RNA-sequencing analysis. Among these genes, we chose four genes (*COL1A1*, *COL1A2*, *COL25A1*, *COL19A1*) involved in the biological process emerged as most altered between the two conditions (Supplementary Figure S1; Figure 4A–I).

All DE genes with their fold change and adjusted *p*-values are listed in Supplementary Figure S2_Total DE genes. A list of the top ECM-related genes is reported in Supplementary Figure S2_Top ECM DE genes.

Among the validated genes, *MKX*, *CADPS*, *THBS4*, and various collagens were found to be upregulated in the RNA-seq analysis. *MKX* is an IRX family-related homeobox protein potentially involved in cell adhesion. *CADPS* encodes a cytosolic and peripheral membrane protein exclusive to the nervous and endocrine systems, required for Ca^2+^-regulated exocytosis of secretory vesicles. *THBS4* encodes thrombospondin proteins, a family of adhesive glycoproteins mediating cell-to-cell and cell-to-matrix interactions. RT-qPCR confirmed the upregulation trend of these three factors (Figure 4A–C). Conversely, *FKBP5* and *UCP3* were downregulated according to the RNA-seq data. *FKBP5* encodes an immunophilin protein involved in immunoregulation and essential physiological functions such as protein trafficking and folding. *UCP3* is part of the mitochondrial anion carrier proteins (MACP) family. RT-qPCR confirmed the downregulation of these genes (Figure 4D,E).

Members of the collagen family were the most deregulated genes identified in the pathway enrichment analyses (Figure 2a,b), showing strong upregulation in transcriptomic data. Among them, *COL25A1* encodes a brain-specific membrane-associated collagen. The encoded protein CLAC (collagenous Alzheimer amyloid plaque component), a proteolytic product, binds amyloid beta-peptides present in Alzheimer’s plaques and inhibits amyloid fibril elongation. *COL19A1* encodes the alpha chain of type XIX collagen, a member of the FACIT collagen family (fibril-associated collagens with interrupted helices). Other members of this family, found in association with fibril-forming collagens like types I and II, help preserve extracellular matrix integrity, though their precise functions remain unknown. *COL1A1* and *COL1A2* encode the pro-alpha 1 chains of type I collagen, which forms a triple helix comprising two alpha1 and one alpha 2 chains. Type I collagen is prevalent in bone, cornea, dermis, tendons, and most connective tissues. Mutations in these genes cause symptoms, with *COL1A1* mutations typically being more severe, reflecting the distinct role of alpha 2 chains in matrix integrity. RT-qPCR highlighted significant upregulation of all collagen family members.

Lastly, since the studied pathologies primarily affect the muscular system, we listed the main deregulated genes involved in skeletal muscle contraction and structure, along with their log_2_FC, *p*-values, and adjusted *p*-values, in Table 2.

## 3. Discussion

Several skeletal muscle adaptations occur through transcriptional programs that modulate gene expression. RNA plays an important role in muscle physiology, indicating that post-transcriptional mechanisms have important regulatory functions in skeletal muscle biology [24]. In the proposed study, we used RNA-seq data from skeletal muscle biopsies of DMD and BMD patients to investigate the transcriptomic changes occurring between the two conditions, aiming to identify a molecular divergence, which could explain the different course of these two pathologies. In 2020, Capitanio and colleagues performed the same comparison based on proteomic analyses between DMD and BMD patients [26]. They showed the minor ECM remodeling involvement for BMD patients compared to DMD group, a greater ability to maintain the mechano-transduction signaling with reduced changes in cytoskeletal and contractile proteins and fewer metabolic changes. In accordance with these results, our analyses on RNA dysregulation highlighted the divergence in gene expression patterns between DMD and BMD patients, showing a clear separation between the two disease conditions. In particular, our work focused on the role of fibrotic processes and ECM dysregulation as a primary factor of divergence in the course and severity of the two dystrophic forms. This is critical because the two diseases share the same genetic basis and differ in the degree of severity at the phenotypic level, at the molecular level, and in gene expression profiles, and can be considered strongly divergent.

### 3.1. Primary Deregulated Pathways: Collagen, ECM Component, and Fibrotic Processes

From pathway enrichment analysis and GO-enriched terms for the biological processes, cellular component, and molecular function, patients with DMD compared to those with BMD showed a particular activation of genes involved in collagen synthesis and extracellular matrix organization. As already described and demonstrated [26], this suggests that a more severe phenotype in patients with DMD than those with BMD may be due to greater deregulation of those pathways, which contribute to impaired contraction, failed muscle regeneration, and misregulated inflammatory response, reflecting the clinical picture of patients observed [1].

What primarily emerged from the study, as an extremely characterizing feature of the DMD condition compared with BMD is the upregulation of all the ECM-related components. In fact, among the most deregulated genes, we detected a total of 20 coding genes for different collagen subtypes (Supplementary Figure S3). This result correlates with the dystrophin protein that plays a key role in maintaining the integrity skeletal muscle, flexibility, and stability of the sarcolemma by anchoring the intracellular actin cytoskeleton to the ECM through the DGC [8]. Dystrophin deficit leads to weakened sarcolemma, functional ischemia, free radical-associated damage, cytosolic calcium overload, failure of muscle regeneration, and muscle damage, but also cardiomyopathy and cognitive manifestations [11]. On the other hand, the described finding is interesting when considered as derived from a direct comparison between patients with DMD and those with BMD, without involving a healthy control group. The aspect we most want to emphasize is that such a large deregulation of ECM components in patients with DMD patients compared to those with BMD may be one of the major causes explaining the different severity degree and faster pathological degeneration in patients with DMD. The regenerative capacity of myofibers is mostly impaired in DMD, possibly as a result of the exhaustion of satellite cells (muscle stem cells) brought on by chronic injury, despite the remarkable ability of skeletal muscle to regenerate in response to injury [27]. Moreover, in the context of ECM remodeling and fibrotic processes, it has also been widely demonstrated that fibro-adipogenic progenitors (FAPs) act as important modulators of homeostasis and skeletal muscle regeneration. Under pathological conditions, as in dystrophic muscles, fibro-fatty infiltration occurs due to dysregulation of these cells [21,28]. Numerous diseases affecting tissues, including DMD, with compromised regeneration mechanisms might involve FAPs as the primary cell-driver of skeletal muscle fibrosis [29]. Some researchers showed that following their rapid expansion in response to damage, FAPs release pro-myogenic factors to promote muscle development and deposit ECM components to replace the injured matrix [30]. FAPs quickly undergo apoptosis and revert to their preinjury levels, which stops ECM deposition and permits complete muscle regeneration. In fibrotic circumstances, FAPs become resistant to apoptosis, staying at persistently high levels and constantly building extracellular matrix, which causes fibrosis [31].

If we now consider the two diseases as models for the analysis of dystrophinopathies in general, it emerges that the different severity degree among these pathologies is primarily sustained and strictly dependent on the level and the type of dysregulation and failures in the ECM components. This aspect draws attention to the fact that targeting the ECM, the enzymes that remodel it, and the receptors that transduce their signals offers promising therapeutic opportunities for many diseases, including DMD and BMD at different levels [32]. Several approaches have been proposed to target MMPs as major altered components in the ECM, including genetic methods to reduce *MMP-9* overexpression in DMD and stem cell-based transplantation of satellite cells to restore dystrophin [33]. In the context of our data, a considerable relevance is given to the possibility of using the deregulated elements of ECM as biomarkers of different dystrophinopathies. For example, serum levels of MMP-9 and TIMP1 (but not osteopontin) have been demonstrated to be significantly higher in DMD patients compared to healthy individuals [34]. In accordance with these observations, we identified the upregulation of metalloproteases and their inhibitors (*MMP2*, *MMP17*, *MMP16*, *MMP14*, *MMP19*, *MMP23b*, *MMP9*, *TIMP1* and *TIMP2*) (Supplementary Figure S4) in patients with DMD compared to the BMD group. Many studies have also demonstrated ECM alteration (MMP/TIMP deregulation) and fibrotic processes in different experimental models, as in the DMD murine model [18,19,20]. Although MMP-ECM interaction can promote ECM degradation and fibrosis regression, ECM continues to be deposited as fibrosis progresses, making the situation worse and possibly irreversible. In fact, MMPs are able to degrade the excessive ECM deposition in fibrotic condition but also to disrupt the normal structure of the ECM itself, resulting in tissue damage and aggravated fibrosis [35]. In some contexts, it has been proposed that the inhibition of MMP-9 activity, which was upregulated in our patients with DMD, may reduce macrophage recruitment and infiltration by going on to reduce fibrosis degree [36]. Overall, if we define fibrosis as the excessive or unregulated deposition of extracellular matrix components themselves, our study allows us to hypothesize that excessive collagen production and consequently excessive ECM accumulation leads to much more pronounced and rapid fibrotic remodeling in patients with DMD than those with BMD [37].

An additional interesting element emerging from this study is related to transforming growth factors (*TGF-β3* and *TGF-α*) and several immune-related genes (e.g., *TREM2*, *C7*, *SPP1*, *FCER2*, *CD24*), which were found to be upregulated in DMD patients compared with BMD subjects. Immune cells play an important role in skeletal muscle homeostasis, repair, and regeneration. However, in DMD, where there is chronic injury due to muscle membrane instability, inflammation becomes chronic and hyperactivated throughout the muscle [16]. A vicious series of events begins, in which inflammation stimulates further pro-inflammatory cytokine signaling, leading to immune infiltration into the muscle and creating a fibrotic and rigid muscle environment. It has already been established that chronic inflammation in DMD results in the pathogenesis of the disease, so our data are in line with what others have already seen [38]. Inflammatory cells detect and eliminate degenerated and damaged muscle cells. T lymphocytes and macrophages comprise most of these infiltrates in young patients with DMD (2–8 years) [39]. M1 macrophages, which act as pro-inflammatory agents, stimulate muscle cell lysis in the early stages of inflammation by producing NOS. These are replaced in the later stages by M2 macrophages, which act as anti-inflammatory agents and facilitate fibrosis and regeneration [40]. In the early stages of the disease, the degenerated muscle regenerates; however, in the later stages, due to reduced regenerative capacity and overexpression of *TGFβ*, the decreased muscle cells are replaced by fibrotic and fatty tissues [41]. Specifically, TGF is released from skeletal muscle upon injury or inflammation, activating myofibroblasts and causing overproduction of ECM, mainly type I and type III collagen, both strongly upregulated in our DMD patients [17]. In addition, CTGF, a nonstructural regulatory protein present in the ECM that plays an important role in fibrosis, is also found to be upregulated in our data. CTGF has the ability to reproduce or amplify the effects of TGFβ on fibrosis. CTGF is associated with virtually all fibrotic remodeling. Furthermore, overexpression of *CTGF* in the muscle of WT mice induces severe fibrosis [42]. Together, these results suggest that both TGFβ and CTGF play a negative role in muscular dystrophies, worsening the DMD condition compared to the BMD clinical picture, by directly inducing fibrotic processes and inhibiting myogenesis [43].

#### ECM Dysregulation Impact, Regeneration Failure, and Signaling Pathways Regulating ECM Genes

Dystrophin absence or malfunction causes sarcolemma instability and myofiber degeneration through a cascade of degenerative events, including chronic inflammation, ECM remodeling, and ultimately, replacement of functional muscle tissue with fibro-adipose tissue [37]. In healthy muscles, ECM provides structural support, mediating cell-to-cell communication and tissue regeneration. Following acute injury, ECM undergoes dynamic remodeling to support tissue repair. In dystrophic patients, such as those with DMD and BMD, continuous muscle damage and ineffective regeneration lead to dysfunctional alteration [44]. These pathological mechanisms lead to excessive ECM deposition, primarily collagens, followed by other proteins such as fibronectin or proteoglycans; tissue stiffness increases with consequent impediment of muscle function and limited force transmission. Moreover, ECM dystrophic remodeling results in the formation, by fibrotic tissue, of a physical barrier, impeding neovascularization, reinnervation, and myoblast migration—all processes essential to effective muscle regeneration [45].

Among the differentially expressed genes from our RNA-seq analyses, we can recognize several key signaling pathways aberrantly activated in DMD and BMD patients at different levels, responsible for pro-fibrotic ECM remodeling. TGF-β is considered a “master regulator” of fibrotic processes, promoting ECM preservation by enhancing protein synthesis such as COL1A1 and COL1A2, and promoting the expression of pro-fibrotic genes like *CTGF*. Elevated TGF-β1 levels are observed in both the plasma and muscle of DMD patients and correlate with fibrosis severity [46]. TGF also suppresses ECM degradation by inhibiting matrix metalloproteinases (MMPs). TGF-β arises as a potential therapeutic target since it has been demonstrated that its inhibition in mouse models reduces fibrosis and improves muscle function [47].

All the pathological ECM changes in patients with DMD and BMD are driven by a complex interplay of multiple cell types such as FAPs, macrophages, satellite cells, and myoblasts [48]. In dystrophic muscles, FAPs are activated by chronic inflammation and pro-fibrotic factors such as TGF-β, which lead to their proliferation and differentiation into myofibroblasts, responsible for the synthesis and abnormal deposition of ECM components (e.g., collagens) [49]. Chronic damage of muscles in DMD patients also impacts macrophage activity. In fact, M2 macrophages contribute to fibrosis by releasing pro-fibrotic cytokines and growth factors, including TGF-β itself, and by promoting ornithine production, which boosts collagen deposition [50].

Dystrophin is also essential for the asymmetric division of muscle stem cells (satellite cells) and the formation of committed myogenic progenitors. In DMD patients, the regenerative capacity of satellite cells is defective. The fibrotic ECM inhibits myoblast differentiation and proliferation, limiting muscle’s ability to regenerate functional fibers [51,52].

Dysregulation of the ECM can significantly impact the management of DMD and BMD. ECM components and their regulators offer potential biomarkers for understanding disease progression and treatment response. Increased levels of collagen type I, III, and VI transcripts could indicate active fibrosis. Imbalances in specific MMPs and TIMPs in serum or muscle biopsies could reflect ECM degradation and remodeling. Elevated CTGF levels could indicate pro-fibrotic activity [53]. Soluble forms of TGF-β or its activation markers could indicate active fibrotic signaling. Other ECM-associated proteins, such as fibronectin and laminin, could also be explored as biomarkers. Additionally, certain muscle-specific miRNAs (dystromirs) are elevated in the serum of DMD and BMD patients and can reflect muscle damage and potentially ECM remodeling [54].

### 3.2. Secondary Deregulated Pathways: Synaptic Organization, Platelet Activation, and Inflammation

Alongside abnormalities in ECM and fibrotic processes, other pathways emerged from the analysis as deregulated when comparing patients with DMD and those with BMD. In fact, numerous elements related to synaptic organization, axonal development, and neuron–neuron communication were found to be deregulated in the gene ontology analysis. Along with pre- and post-synaptic abnormalities, research shows that the neuromuscular junction (NMJ) is more vulnerable to damage from contractions, which results in functional alterations in neuromuscular transmission and nerve-evoked electromyographic activity. These observations imply that changes in dystrophic muscle’s NMJ may contribute to muscle weakness by impairing neuromuscular transmission [55].

Another deregulated element emerged from gene ontology enrichment analysis was related to platelet activation and function [56]. Numerous studies on coagulation difficulties in DMD have been reported; these include bleeding complications, particularly prolonged bleeding during scoliosis surgery, as well as thromboembolic events [57]. Although DMD patients do not frequently experience spontaneous bleeding diathesis, the recent rise in life expectancy and the increased incidence of major surgery highlight the need for a more thorough understanding of hemostasis in DMD. Additionally, when heart failure occurs, DMD patients are frequently given anticoagulants as antithrombotic prophylaxis [58].

Finally, the inflammatory response has emerged from gene ontology evaluation of DMD and BMD patients. In fact, we found several inflammation-related genes deregulated, such as those in the Oncostatin M pathway (e.g., *CCL14*, *PTGFR*, *SERPINA3*, *CCL13*, *CSF1R*, *CEBPB*, *NRROS*, *TNFAIP6*). It is commonly accepted that self-maintaining inflammatory responses worsen muscle degeneration: a vicious cycle in the muscle tissue is created by cellular necrosis brought on by the lack of dystrophin, followed by inflammation and excessive fibrosis [59]. DMD models exhibit dysregulation of inflammatory signaling pathways, including nuclear factor kappa B (*NF-κB*) or its downstream mediators, including tumor necrosis factor alpha (*TNF*α), interleukin-6 (*IL-6*), and interleukin-1 beta (*IL-1β*) [60]. To promote immune cell infiltration and function, satellite cells have been shown to release a panel of pro-inflammatory cytokines, including IL-1, IL-6, and TNF-*α*. Supporting this, our results show several of these cytokines upregulated in DMD patients compared to BMD (e.g., *IL17B* FC: 2.26, *IL1RL1* FC: 1.42, *TNFRSF18* FC: 2.27), indicating a more severe inflammatory condition in DMD subjects.

Immune cells then release a multitude of diffusible substances, including globular adiponectin, growth factors, ECM constituents, and MMPs involved in ECM remodeling. As happens with muscle aging or muscular dystrophies like DMD, changes in quality, quantity, and timing result in decreased regeneration, increased muscle wasting, and deposition of fibrotic tissue [61].

## 4. Materials and Methods

### 4.1. Patient Enrollment

Patients presenting with early clinical onset, markedly elevated creatine kinase (CK) levels, out-of-frame mutations, and—when muscle biopsy was performed—less than 5% revertant fibers detected by immunofluorescence (IF) and absence of dystrophin, were classified as having Duchenne muscular dystrophy (DMD). Patients exhibiting absent or very mild clinical symptoms, moderately elevated CK levels, in-frame mutations, and—if biopsy was performed—dystrophin presence detected by IF but with altered Western blot results, were classified as having Becker muscular dystrophy (BMD) (Table 3).

### 4.2. Muscle Biopsy Isolation

Open muscle biopsies were obtained from the quadriceps muscle (typically the left quadriceps) after informed parental consent. Muscle samples were oriented and rapidly frozen in isopentane cooled by liquid nitrogen. Portions of the sample were used for diagnostic purposes, while the remainder was stored in liquid nitrogen for potential further studies, as explained to the parents. Stored samples were assigned serial numbers corresponding to each patient.

### 4.3. Blood DNA Isolation, Muscle Sample Homogenization, and RNA Extraction

Genomic DNA was extracted from blood samples using the Maxwell^®^ 16 Blood DNA Kit (Promega, Milan, Italy) for genetic analyses. Multiplex Ligation-dependent Probe Amplification (MLPA) with P034 and P035 Salsa kits (MRC-Holland) was used to detect deletions and duplications. For patients negative by MLPA, whole-exome sequencing (WES) was performed using the Human Core Exome kit (Twist Bioscience, San Francisco, CA, USA) on an Illumina NovaSeq 6000 system.

Approximately 15 mg of cryopreserved skeletal muscle tissue was homogenized using the CK14 Precellys lysing kit (Bertin Technologies, Montigny-le-Bretonneux, France). Total RNA was extracted from muscle biopsies using TRIzol^®^ reagent (Life Technologies, Carlsbad, CA, USA), following the manufacturer’s protocol. Briefly, 200 µL of chloroform was added to homogenates, which were centrifuged at 12,000× *g* for 10 min at 4 °C. The aqueous phase was recovered, mixed with 500 µL isopropanol, and centrifuged again under the same conditions. Pellets were washed with ethanol, centrifuged at 7600× *g* for 5 min, air-dried, resuspended in nuclease-free water, and quantified. RNA quality was assessed with a Nanodrop ND-100 spectrophotometer and the Agilent 4200 TapeStation system, yielding RNA Integrity Numbers (RINs) between 5.9 and 9.6.

### 4.4. RNA-Seq Library Preparation and Bioinformatic Data Analysis

cDNA libraries were prepared starting from 500 ng of total RNA and using Illumina TruSeqStranded RNA Library Prep, version 2, Protocol D (Illumina, San Diego, IL, USA) by Biomek i7 Automated Workstation. The quality of each library was assessed by 4200 Tape Station System using a “DNA High sensitivity” assay (Agilent, Santa Clara, CA, USA). Libraries were fluorometrically quantified using a High Sensitivity dsDNA assay with a Qubit device (Life Science Technologies, Waltham, MA, USA). The sequencing step was performed with NGS technologies using Illumina Genome Analyzer and the NextSeq 500/550 High Output v2.5 kit (150 cycles) (Illumina, San Diego, IL, USA), processed on Illumina NextSeq 550.

FastQ files were produced using Illumina’s bcl2fastq2 program, using version 2.17.1.14. available online (https://support.illumina.com/sequencing/sequencing_software/bcl2fastq-conversion-software.html (accessed on 28 January 2025)), starting from raw sequencing reads produced by the Illumina NextSeq sequencer. For each sample, the number of coding and non-coding RNA transcripts was counted and assessed independently. Gene and transcript intensities were computed using the “stranded” option and the reference genome GRCh38 (Gencode release 27) in the STAR/RSEM software [62] and R package DeSeq2 was employed for RNA differential expression analysis [63,64]. Data were not corrected for batch effect considering that each sample had the same source and sampling and storage conditions, and were run in a single NextSeq cartridge. The workflow, including quality control filter, trimming of adapters, and read mapping, was implemented in the Docker4Seq package. For further analysis, count threshold was set at 1,000,000; transcripts with |log_2_FoldChange (DMD sample/BMD sample)| ≥ 1 and FDR ≤ 0.1 were maintained as differentially expressed [65,66].

### 4.5. Pathway Analysis

Gene enrichment analysis was performed on coding genes [67]. A Gene Ontology (GO) analysis was conducted for biological processes, cellular components, and molecular function, while BIOPLANET and REACTOME pathway analysis derived from enrichR (Kyoto Encyclopedia of Genes and Genomes available online; accessed on 28 January 2025) was conducted for pathway analysis. For this investigation, we used the enrichR web tool (https://maayanlab.cloud/Enrichr/) (accessed on 28 January 2025/) [68,69].

### 4.6. Real-Time PCR

Using NCBI-provided human gene sequences (www.ncbi.nlm.nih.gov/nucleotide (accessed on February 2025)), PCR oligonucleotide for gene pairs were selected using online Primer3plus (https://www.bioinformatics.nl/cgi-bin/primer3plus/primer3plus.cgi (accessed on February 2025))(primers are listed in Table 4). cDNAs were prepared using iScriptTM Reverse Transcription Supermix for RT-qPCR (Bio-Rad, Milan, Italy) starting from 500 ng of total RNA. For the reaction mix, 200 nM of each oligonucleotide, 7.5 µL of iQ SYBR Green Supermix (Bio-Rad, Milan, Italy), and 1 µL of cDNA template (or water control) were used. The reaction was performed using the CFX Connect™ Real-Time PCR Detection System (Bio-Rad, Milan, Italy). Cycle threshold (Ct) values were automatically recorded for each replicate qPCR reaction, and mean Ct values were normalized against those determined for *GAPDH*. Fold-expression differences were determined using the 2^−ΔΔCt^ method. The results were considered statistically significant with a *p*-value < 0.05.

### 4.7. Statistical Analyses

GraphPad PRISM version 9 (SD, USA) was used to perform statistical analysis and produce graphs. Each analysis has been evaluated in technical duplicate and each patient represents a biological replicate for the specific pathological condition. Data were tested for normality by applying the D’Agostino and Pearson test, Anderson–Darling test, Shapiro–Wilk test, and Kolmogorov–Smirnov test (all passed). Statistical analysis was performed using the Two-tailed Unpaired Student’s t-test; data were expressed by means ± SEM and deemed statistically significant when the p-values were less than 0.05.

## 5. Conclusions

Our findings demonstrate that Duchenne and Becker muscular dystrophies differ molecularly, notably in gene expression intensity. We identified molecular distinctions between the two phenotypic groups, highlighting pathways that likely contribute to the more severe DMD phenotype. Compared to patients with BMD, patients with DMD exhibited upregulation of genes involved in collagen synthesis and extracellular matrix (ECM) organization, key contributors to fibrosis. This suggests that increased dysregulation of these pathways underlies the more severe clinical manifestations in DMD. Furthermore, pro-fibrotic factors such as TGF-β, CTGF, and MMPs were elevated in DMD samples, supporting the role of fibrosis in disease severity. Understanding ECM dysregulation may enable patient stratification for targeted anti-fibrotic therapies, with those exhibiting pronounced fibrosis—assessed via imaging or biomarkers—potentially benefiting most. Identifying individual pro-fibrotic pathways can guide personalized treatment selection, while stratification by age and disease stage could permit earlier intervention. ECM-related biomarkers also hold promise as prognostic tools and predictors of therapeutic response, including to dystrophin-restoring treatments. High baseline pro-fibrotic markers or extensive fibrosis may indicate rapid progression and poorer outcomes, while biomarker monitoring can provide early evidence of treatment efficacy.

Overall, this study offers preliminary insights into gene expression differences between DMD and BMD, laying the groundwork for identifying mechanistic targets to inform personalized therapies.

## 6. Limitations of the Study

This study was limited by a small patient cohort, reflecting the challenges of obtaining muscle biopsies from pediatric muscular dystrophy patients. Additionally, Italian legislation restricts muscle biopsy collection from healthy individuals, limiting access to control samples.

Future work aims to expand the cohort by including biopsies from biobanks and incorporating healthy controls. This would enable better characterization of pathological versus physiological conditions and validation of current findings, advancing under-standing of common and distinct pathological factors.

## Figures and Tables

**Table 1:** Number of differently expressed genes based on gene biotype and deregulation.

Differentially Expressed Genes		
	Protein Coding	Non-Coding
Upregulated	1455	268
Downregulated	354	138
Subtotal	1809	406
Total	2215	

**Table 2:** Deregulated skeletal muscle genes, encoded protein, function (https://www.genecards.org/) (accessed on 15 February 2025) and relative Log_2_FC, *p*-value, and adj *p*-value.

Gene	Log_2_FC	*p*-Value	Adj *p*-Value	Protein	Function in Muscles
*MYH1*	−1.700586668	0.000746513	0.004671804	Myosin Heavy Chain 1	Major contractile protein, it converts chemical energy into mechanical energy through the hydrolysis of ATP.
*MYH14*	−1.122451416	1.52 × 10^−9^	0.000000088	Myosin Heavy Chain 14	Represents a conventional non-muscle myosin. It should not be confused with the unconventional myosin−14 (MYO14).
*MYH11*	1.028496227	0.0174439	0.059487717	Myosin Heavy Chain 11	Smooth muscle myosin. It functions as a major contractile protein, converting chemical energy into mechanical energy through the hydrolysis of ATP.
*MYH8*	2.268592775	0.001163423	0.006689683	Myosin Heavy Chain 8	Class II or conventional myosin heavy chains. Functions in skeletal muscle contraction.
*MYH3*	2.926975986	5.90 × 10^−8^	0.000001801	Myosin Heavy Chain 3	Major contractile protein actin-associated.
*ACTA2*	1.032325389	0.000988763	0.005874898	Actin Alpha 2	Smooth muscle actin that is involved in vascular contractility and blood pressure homeostasis.
*ACTN1*	1.147716074	1.29 × 10^−5^	0.000158151	Actinin Alpha 1	Skeletal muscle isoforms are localized to the Z-disc. They help anchor the myofibrillar actin filaments.

**Table 3:** Clinical and demographic data of DMD and BMD cohorts.

Code	Dob	Diagnosis	Age at Muscle Biopsy (Mnts)	Gene Variants	Age of Onset (Mnts)	Disease Duration (Yrs)	Ambulatory or Not	Age at Evaluation (Yrs)	NSS	6MWT (cm)	FVC (%)
B1	1997	Bmd	29	Ex. Del. 47–52	14	26	Amb.	17	34	609	1
B2	1997	Bmd	32	Ex. Del 48	18	25	Amb.	17	34	614	0.93
B3	2000	Bmd	46	Ex. Del 45–47	17	22	Amb.	17	29/34	475	1
B4	2001	Bmd	122	Ex. Del. 45–47	23	21	Amb.	16	33	614	1
B5	2005	Bmd	92	Ex 6: c.358G > T*p*.120Vl > Phe	72	13	Amb.	16	34	753	1
B6	2006	Bmd	95	Ex. Del. 48–50	22	17	Amb.	14	34	682	1
D1	2002	Dmd	28	Ex. Del 46–49	19	21	Not amb. from 16 yrs	15	21	232	1
D2	1993	Dmd	136	Ex. Dup. 19	49	27	Amb. + deficit*	17	21	360	NA
D3	2006	Dmd	20	Stop point mut. Ex. 70: c.10141C > Tp.Arg3381X	13	18	Amb. + deficit*	17	21	449	0.78
D4	2006	Dmd	17	Ex. Del. 46–52	15	16	Not amb. from 13 yrs	12	2	246	0.77
D5	2005	Dmd	36	Ex. Dup. 1–9	19	17	Not amb. from 16 yrs	7	15	475	NA
D6	2004	Dmd	56	Ex. Del. 3–27	23	18	Amb. + deficit*	8	31	514	NA
D7	2008	Dmd	17	Ex. Del. 3–26	3	16	Amb. + deficit*	16	22	359	1
D8	2003	Dmd	86	Point mutation Int.5: c.358–1G > A	29	19	Not amb. from 14 yrs	13	18	246	0.95
D9	2008	Dmd	27	Ex. Del. 8–12	25	14	Not amb. from 15 yrs	11	11	302	NA
D10	2006	Dmd	57	Ex. Del. 49–54	60	13	Not amb. from 14 yrs	13	12	247	NA
D11	2008	Dmd	54	Ex. Dup. 62–67	53	12	Not amb. from 13 yrs	12	13	186	1
D12	2008	Dmd	56	Ex. Del. 3–29	36	13	Not amb. from 7 yrs	5	14	201	NA

Amb: Ambulatory. Amb. + deficit* = patients not available in follow up study. NSS = North Star Scale. 6MWT = 6-Minute Walk Test. FVC (%) = Forced Vital Capacity (%).

**Table 4:** Primers sequences for RT-qPCR.

GENE SYMBOL	PRIMER FORWARD	PRIMER REVERSE	AMPLICON LENGTHS
*MKX*	CCTTACAGGCATGAAGGGGG	GTGGTGCTTTCCAACAGTGC	73 bp
*CADPS*	TGCAGAAAATGTAGGCCGGT	CGTGGTGCTCCTCATTTTGC	107 bp
*THBS4*	AACCCAGAGCTGAACCCTTG	ACACACATGTCACATCCCCC	73 bp
*FKBP5*	GGACTGGACAGTGCCAATGA	GGCACATGGAGATCTGCAGT	151 bp
*UCP3*	AGCCCCCTCGACTGTATGAT	CCCAAACGCAAAAAGGAGGG	89 bp
*COL25A1*	CCAAAATCGCCTCTCCCGAT	TTGGCACAGATTGTCCCAGT	55 bp
*COL19A1*	CCTTACAGGCATGAAGGGGG	TCCCATGGAGCCCTTGTTTC	64 bp
*COL1A1*	CCTGGGGCAAGACAGTGATT	TCGAAGCCGAATTCCTGGTC	109 bp
*COL1A2*	CTGGTAGTCGTGGTGCAAGT	AGGACCTTCTTTTCCAGCGG	143 bp
*GAPDH* (HG *)	AGGAGTAAGACCCCTGGACC	GGGGAGATTCAGTGTGGTGG	113 bp

* HG = Housekeeping Gene.

## Data Availability

The RNA-sequencing datasets for this study can be found in the GEO repository (GSE291383) and the Zenodo repository (DOI:10.5281/zenodo.15267052).

## Supplementary Materials

The following supporting information can be downloaded at: https://www.mdpi.com/article/10.3390/ijms26146594/s1.
