# Supplementary material for: Comparative Transcriptomic Profiling in Patients Affected by Duchenne and Becker Muscular Dystrophies: A Focus on ECM Genes Dysregulation
Source: Int J Mol Sci. 2025 Jul 9;26(14):6594. doi: 10.3390/ijms26146594 (PMC12294368; doi:10.3390/ijms26146594)
Supplement: Supplementary file 1 [file ijms-26-06594-s001.zip › Supplementary file 1_Reviewed.pdf]

Supplementary Figure S1

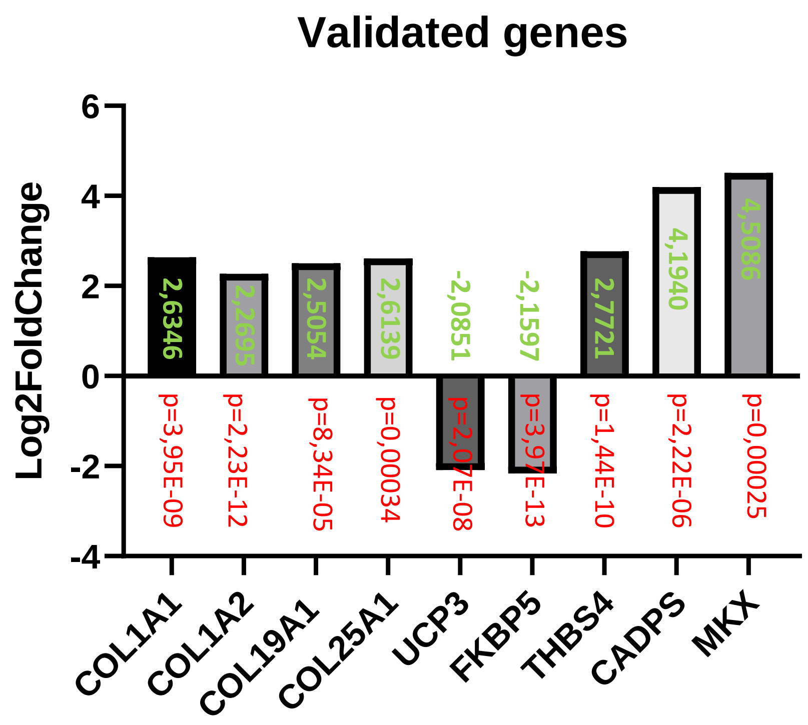

Supplementary Figure S1 (.pdf): Bar Plot. Supplementary file 1 shows Log2FC (green) and p-value (red) from RNA-seq analysis of validated genes. RT-qPCR data can be found in the main text, Figure 4.
