# Supplementary material for: Comparative Transcriptomic Profiling in Patients Affected by Duchenne and Becker Muscular Dystrophies: A Focus on ECM Genes Dysregulation
Source: Int J Mol Sci. 2025 Jul 9;26(14):6594. doi: 10.3390/ijms26146594 (PMC12294368; doi:10.3390/ijms26146594)
Supplement: Supplementary file 1 [file ijms-26-06594-s001.zip › Supplementary file 4_Reviewed.pdf]

Supplementary Figure S4

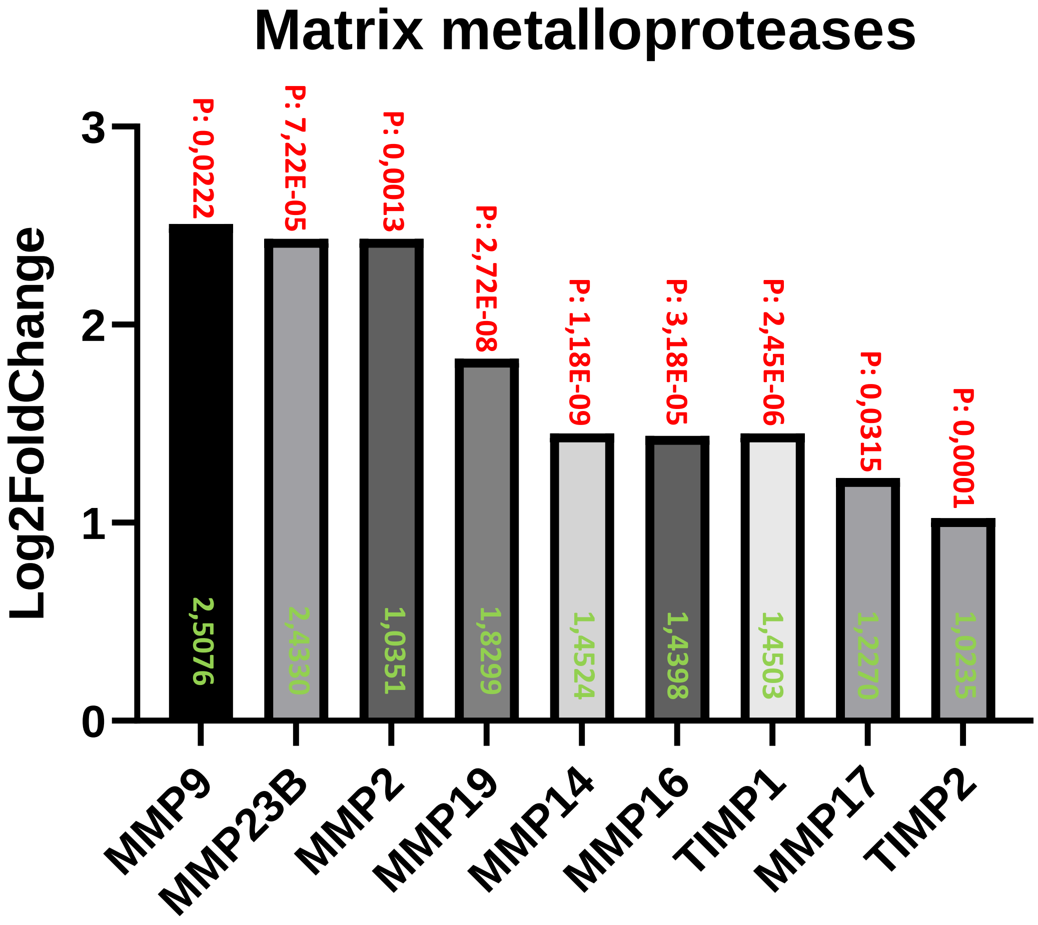

Supplementary Figure S4 (.pdf). Bar Plot. Supplementary Figure 4 shows Log2FoldChange (green) and p-value (red) of the main deregulated genes involved in extracellular matrix alteration.
